# Hair follicle-derived mesenchymal stem cells decrease alopecia areata mouse hair loss and reduce inflammation around the hair follicle

**DOI:** 10.1186/s13287-021-02614-0

**Published:** 2021-10-21

**Authors:** Weiyue Deng, Yuying Zhang, Wei Wang, Aishi Song, Omar Mukama, Jiarong Huang, Xiaobo Han, Sihao Deng, Zuoxian Lin, Jean du Dieu Habimana, Rongqi Huang, Kexin Peng, Bing Ni, Shusheng Zhang, Xiaoxin Yan, Ji Li, Lin-Ping Wu, Zhiyuan Li

**Affiliations:** 1 Department of Anatomy and Neurobiology, Xiangya School of Medicine, Central South University, Changsha, China; 2 CAS Key Laboratory of Regenerative Biology, Guangdong Provincial Key Laboratory of Stem Cell and Regenerative Medicine, Guangzhou Institutes of Biomedicine and Health, Chinese Academy of Sciences, Guangzhou, China; 3 Center for Chemical Biology and Drug Discovery, Guangzhou Institute of Biomedicine and Health, Chinese Academy of Sciences, Guangzhou, China; 4 NHC Key Laboratory of Birth Defect for Research and Prevention, Hunan Provincial Maternal and Child Health Care Hospital, Changsha, China; 5 Changsha Stomatological Hospital, Changsha, China; 6 Bioland Laboratory, Guangzhou, China; 7 GZMU-GIBH Joint School of Life Sciences, Guangzhou Medical University, Guangzhou, China

**Keywords:** Alopecia areata, Hair follicle-derived mesenchymal stem cells, Stem cell therapy, Hair loss treatment

## Abstract

**Background:**

Alopecia areata (AA) is a common autoimmune hair loss disease with increasing incidence. Corticosteroids are the most widely used for hair loss treatment; however, long-term usage of hormonal drugs is associated with various side effects. Mesenchymal stem cells (MSCs) therapy has been studied extensively to curb autoimmune diseases without affecting immunity against diseases.

**Methods:**

Hair follicle-derived MSCs (HF-MSCs) were harvested from the waste material of hair transplants, isolated and expanded. The therapeutic effect of HF-MSCs for AA treatment was investigated in vitro AA-like hair follicle organ model and in vivo C3H/HeJ AA mice model.

**Results:**

AA-like hair follicle organ in vitro model was successfully established by pre-treatment of mouse vibrissa follicles by interferon-γ (IFN-γ). The AA-like symptoms were relieved when IFN-γ induced AA in vitro model was co-cultured with HF-MSC for 2 days. In addition, when skin grafted C3H/HeJ AA mice models were injected with 10^6^ HF-MSCs once a week for 3 weeks, the transcription profiling and immunofluorescence analysis depicted that HF-MSCs treatment significantly decreased mouse hair loss and reduced inflammation around HF both in vitro and in vivo.

**Conclusions:**

This study provides a new therapeutic approach for alopecia areata based on HF-MSCs toward its future clinical application.

**Supplementary Information:**

The online version contains supplementary material available at 10.1186/s13287-021-02614-0.

## Introduction

The distress caused by hair loss is rising with increasing life pressure and environmental factors. Hair loss is usually associated with abnormal hair follicle cycling and morphology. Stem cells drive the hair cycle from hair follicles growth rest (telogen) to a new growth phase (anagen) [1].

Alopecia areata (AA) is a common non-scarring autoimmune disorder that occurs in genetically susceptible individuals, and it is triggered by some unknown environmental factors [2]. The incidence of this disease scaled up from 0.1% in 1970 to 2.11% in recent years [3, 4]. It has an apparent effect and brings more psychological pressure to the patients than other dermatological disorders [5]. Although its etiology and pathogenesis are still elusive, CD8^+^ T cells infiltration was identified as the major irritant in AA [6, 7]. Hair follicles keep immune privilege by suppression of autoantigen presentation system like major histocompatibility complex (MHC) class I [8]. Some proinflammatory cytokines like tumor necrosis factor-α (TNF-α) and interleukin-6 (IL-6) also coordinate cyclical hair growth within the AA pathogenesis [9–11]. Hormone drugs are the most used topical treatment clinically, though they could exhibit some side effects [12]. Some alternatives treatments of androgenetic alopecia (AGA) have been reported like platelet-rich plasma (PRP) local injection [13], but research in AA is still limited. Therefore, there is a need for the development of alternative therapy in AA.

Mesenchymal stem cells (MSCs) exhibit excellent interaction with immune cells and immunoregulation [14]. MSCs injection can suppress lymphocyte proliferation in some immunopathy like leucoderma and lung injury [15]. A clinical study showed that autologous adipose-derived stromal vascular cells (ADSVCs) injection could treat alopecia areata safely and effectively [16]. Besides, MSCs isolated from hair follicles (HF) have great differentiation potential and are user-friendly [17–19]. The injection of hair follicle stem cells (HFSCs) to a rat model of middle cerebral artery ischemia/reperfusion reduced infarct volume and promoted neurological recovery [20]. Sun et al. group reported that intravenous injection of HF-MSCs to the rats with acute pancreatitis regenerated damaged pancreas and reduced IL-6 and TNF-α in the serum [21]. The pluripotent factor octamer-binding transcription factor 4 generated HF-MSCs can differentiate to enucleated adult-type erythrocytes, providing a new pathway for patient-specific transfusion [22]. Furthermore, engineered HF-MSCs released human insulin in a controlled manner that reversed hyperglycemia in mice with type 1 diabetes [23].

In this study, we established an in vitro AA-like model by using IFN-γ-induced mouse vibrissa follicle. We observed immunotoxicity cytokines suppression and hair shaft elongation in vibrissa follicles after HF-MSC treatment in vitro. The injection of HF-MSCs restrained AA mice hair loss and reduced inflammation around HF. Overall, the finding of this study provides a new stem cell-based therapeutic method for AA treatment.

## Methods

### Isolation and cultivation of human hair follicle mesenchymal stem cells

HF-MSCs were isolated as described previously [18, 24]. Briefly, human hair follicles were obtained from the waste material of an informed hair transplant patient. The hair follicles were drawn off from surrounding tissue by tweezers and washed extensively with Hank's balanced salt solution containing 1% penicillin–streptomycin. After cutting hair shafts, hair follicles were transferred into a 24-well culture plate, with one follicle per well containing Dulbecco’s modified Eagle Medium-Ham F-12 (DMEM/F-12; Gibco, Life, USA) supplemented with 10% fetal bovine serum (FBS; Gibco, Life, USA) and 2 ng mL^−1^ of basic fibroblast growth factor (bFGF; Santa Cruz Biotechnology, USA) at 37 °C with 5% CO2. The culture medium was changed every 3 days. After fibroblast-like cells migration from the hair follicles, cells originating from the dermal sheath or papilla were selected and digested with 0.25% trypsin and then subsequently subcultured in DMEM/F-12 supplemented with 10% FBS until 80% confluency.

### Animals

This study was approved by the Guangzhou Institutes of Biomedicine and Health-Chinese Academy of Science experimental animal center, and all animal housing and handling were carried out according to IACUC guidelines (Permit No. 2018051). The C3H/HeJ mice were purchased from The Jackson Laboratory, USA, and C57BL/6 mice were purchased from Charles River Laboratories, China.

### Isolation and cultivation of hair follicle organ

Mouse vibrissa follicles were isolated from a 6-week-old female C57BL/6 mouse. Animals were anesthetized with CO_2_ and killed by cervical dislocation. The skin around the nose was cut with the aid of a stereomicroscope and pulled up sharply to expose clean vibrissa follicles by using forceps. After extensive washing with phosphate-buffered saline (PBS) containing 100 IU mL^−1^ penicillin, one hair follicle per well was cultured at 37 °C in 5% CO_2_ [25].

Mouse vibrissa follicles were cultured in 48-well plates with HF medium or inflammation-induced medium. HF medium [26]: William's E medium(Gibco, Life, USA) was supplemented with 10 mg mL^−1^ insulin (Sigma, Germany), 10 ng mL^−1^ hydrocortisone (Sigma, Germany), 100 IU mL^−1^ penicillin (Sigma, Germany), 100 mg mL^−1^ streptomycin (Gibco, Life, USA) and 1 mmol L^−1^ l-glutamine (Gibco, Life, USA). Inflammation-induced medium: complete HF medium was supplemented with 100 IU mL^−1^ IFN-γ.

### Hair follicle organ co-culture with HF-MSC

HF-MSCs were seeded for 48 h in advance with a concentration of 10^4^ per well with F12 and 10% FBS in 24-well plates. When mouse vibrissa follicles were cultured with 100 IU mL^−1^ IFN-γ for 4 days, the medium was removed, and the follicles were washed three times using PBS. After HF-MSC reached 80–90% confluency, the medium was removed and rewashed three times by PBS, then 300 µL HF medium was added per well. The mouse vibrissa follicles were then transferred carefully from the 48-well plate to the 24-well plate with HF-MSC and ensured every vibrissa follicles were immersed and floated in the medium.

### Generation of alopecia areata in the C3H/HeJ mouse and treatment with HF-MSC

First, mice were anesthetized using intraperitoneal injection of pentobarbital (ZaoZhuang Water Tailan Chemical, China). Then, transplanted a full-thickness circular piece of lesional skin, approximately 1–1.5 cm in diameter graft, from an older C3H/HeJ mouse that almost total hair loss to recipient C3H/HeJ mice (8-week-old, female) as previously described [25]. After 8–10 weeks, nine recipient mice with severe alopecia in the abdomen and 20% hair loss in the back were selected and divided randomly into two groups: Five mice received MSCs with a concentration of 10^6^ in 300 µL PBS, and four mice received 300 µL phosphate-buffered saline (PBS) intravenously injection weekly, 3 weeks in a row. Six weeks after injection, we grafted the lesion skin and then dipped the skin in 4% paraformaldehyde or liquid nitrogen.

### Flow cytometry

Trypsinized Human HF-MSCs were resuspended in 0.5 mL of PBS and then conjugated with antibodies using Human MSC Analysis Kit (BD Biosciences, USA). The human HF-MSCs were subsequently washed with PBS and resuspended in 4% PFA prior to flow cytometric analysis performed using a FACS Calibur cytometer.

### Real-time PCR

Total RNA was extracted with TRIzol (Invitrogen, USA), and cDNA was transcribed using a cDNA synthesis kit (Vazyme, China). Concentrations of genomic RNA were measured by Nanodrop (Thermo Fisher Scientific, USA). Real-time PCR was performed in a volume of 20× QuantiTect SYBR Green PCR Master Mix 5 µL (Vazyme, China), 0.4 µL forward and reverse mixture primers (5 μM) and 2.6 µL template cDNA. RT-qPCR reactions were performed using a CFX96 Real-Time System (Bio-Rad, USA) at 95 °C for 10 min and followed by 39 cycles at 95 °C for 15 s, 60 °C for 1 min, finally get the melt curve from 65 to 95 °C. The following gene-specific primers were used (forward and reverse sequence): Ki67 (F: 5′-ATCATTGACCGCTCCTTTAGGT-3′; R:5′-GCTCGCCTTGATGGTTCCT-3′), Caspase1 (F: 5′-AATACAACCAC TCGTACACGTC-3′; R: 5′-AGCTCCAACCCTCGGAGAAA-3′), MHC I (F: 5′-ACCAGCAGTACGCCTACG A-3′; R: 5′-AACCAGAACAGCAACGGTCG-3′), TNF-α (F: 5′-CCCTCACACTCAGATCATCTTC-3′; R: 5′-GCTACGACGTGGGCT ACAG-3′), IL-6 (F: 5′-TAGTCCTTCCTACCCCAATTTCC-3′; R: 5′-TTGGTCCT TAGCCACTCCTTC-3′).

### Western blot analysis

Total protein was extracted from twenty mouse vibrissa follicles. Protein concentrations were determined using a Bicinchoninic Acid protein assay kit (Beyotime, China). Briefly, 30 µg of protein was loaded on 7.5–12.5% acrylamide gels for the electrophoretic separation of proteins under denaturing conditions and transferred to PVDF (Bio-Rad, USA), followed by overnight incubation at 4 °C with the corresponding primary antibodies (1:500-diluted Ki67 Rabbit mAb (Cell Signaling Technology, USA); 1:800-diluted Cleaved Caspase-1 Rabbit mAb (Cell Signaling Technology, USA); 1:500-diluted MHC I mouse mAb (Santa, USA) and 1:1000-diluted β-actin Rabbit mAb (Bioss, China). After incubation with peroxidase-conjugated secondary antibodies (Beyotime, China), the bands were visualized using Chemocam imager 6.0 (Intas, Germany). Protein expression levels were normalized to corresponding β-actin levels. Western blots were quantified by Image J (version 1.8.0).

### Immunohistochemical staining

HF-MSCs were fixed in 4% paraformaldehyde for 15 min, washed by PBS, then incubated with 10% goat serum and 0.1% Triton X-100 in PBS 1 h and 1:200-diluted mouse anti-human CD90, CD 44, CD73, CD105, CD31, CD34, CD45 antibody (BD Biosciences, USA) at 4 °C overnight.

Skin or hair follicles organs were fixed in 4% paraformaldehyde. Then, tissues were dehydrated by graded sucrose. Then, the tissues were embedded in an optimal cutting temperature compound (Sakura Finetek, USA) and cut into 10-μm-thick sections by freezing microtome (Leica, Germany).

Tissues sections were incubated at 4 °C overnight with 10% goat serum and 0.1% Triton X-100 in PBS 1 h and 1:400-diluted rabbit anti-ki67 (Cell Signaling Technology, USA), 1:100-diluted rat anti-CD8 (Santa Cruz Biotechnology, USA), 1:200-diluted rabbit anti-cytokeratin 15 antibodies (Abcam, USA), 1:100-diluted mouse SOX9 (Santa Cruz Biotechnology, USA) and 1:500-diluted rabbit anti-Lhx2/LH2 (Abcam, USA).

After primary antibody incubation, the sections and cells were reacted with Alexa Fluor 488 or 594-conjugated anti-rabbit/rat/mouse IgG (Molecular probes, Life, USA) (1:400) for 1 h. 4′-6-diamidino-2-phenylin-dole (DAPI; Beyotime, China) was used to stain the Cell nucleus for 15 min, and then the sections were mounted with an anti-fluorescence quenching agent (Biosharp, China). Representative images were captured using an inverted fluorescence microscope (Leica, Germany). All experimental procedures described above were conducted in a dark room. And fluorescence intensity and areas were analyzed by Image J (version 1.8.0).

### Histological eosin analysis

Tissue sections were stained with hematoxylin–eosin (HE) staining Kit (Beyotime, China). The images were captured on an inverted fluorescence microscope (Leica, Germany).

### Statistical analysis

The data were expressed as Mean ± SD. Prism Graph Pad (version 8.0, La Jolla, CA) was used to perform the statistical analysis. One-way ANOVA and Tukey’s test (analysis of variance) were used to verify the differences between groups. *p < 0.05 or **p < 0.01 was considered statistically significant.

## Results

### Isolation and identification of HF-MSCs

**Fig. 1:**
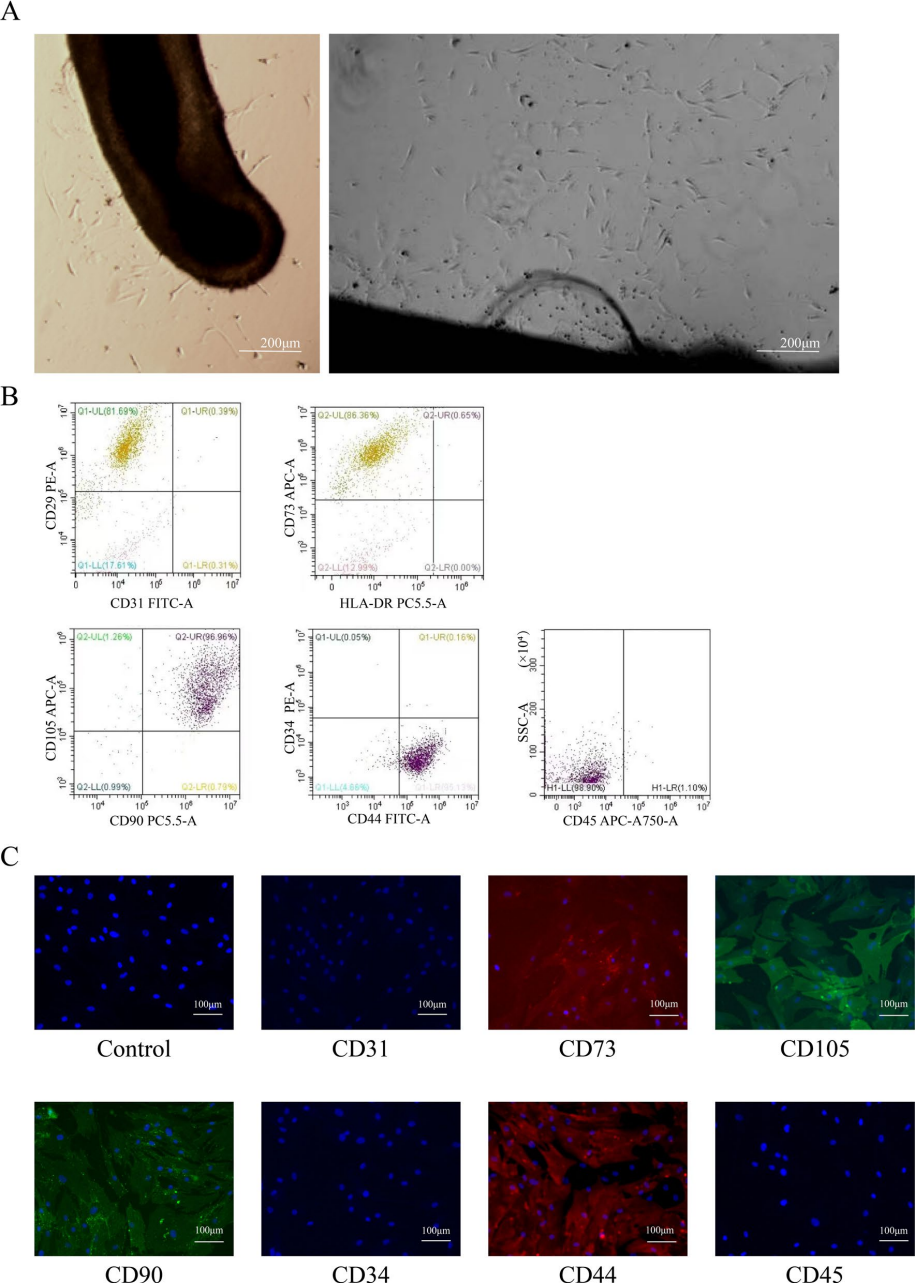
Isolation and characterization of human hair follicle-derived mesenchymal stem cells (HF-MSCs). **A** Cells that migrated from hair follicles and proliferated onto the culture plate exhibited fibroblast-like characteristics (bar = 200 μm). **B** Flow cytometry was used to measure the cell surface expression of MSC biomarkers. The cells expressed CD29, CD44, CD73, CD90, CD105 but not CD31, CD34, CD45, HLA-DR. **C** Immunofluorescence analysis was used to measure the surface markers of HF-MSCs. The cells expressed CD44, CD73, CD90, CD105 but not CD31, CD34, CD45 (bar = 100 μm)

HF-MSCs migrated out from human hair follicles and proliferated onto the culture plate. It displayed a fibroblast-like morphology (Fig. 1A). Flow cytometry assays showed that HF-MSCs expressed CD29, CD44, CD73, CD90, and CD105 while negative control expressed CD24, CD31, CD45, HLA-DR (Fig. 1B). Immunofluorescence staining showed a similar result that CD44, CD73, CD90, and CD105 were highly positive, but negative for CD31, CD34, and CD45 (Fig. 1C).

### Vibrissa follicle can be induced to AA-like symptoms by using IFN-γ treatment

**Fig. 2:**
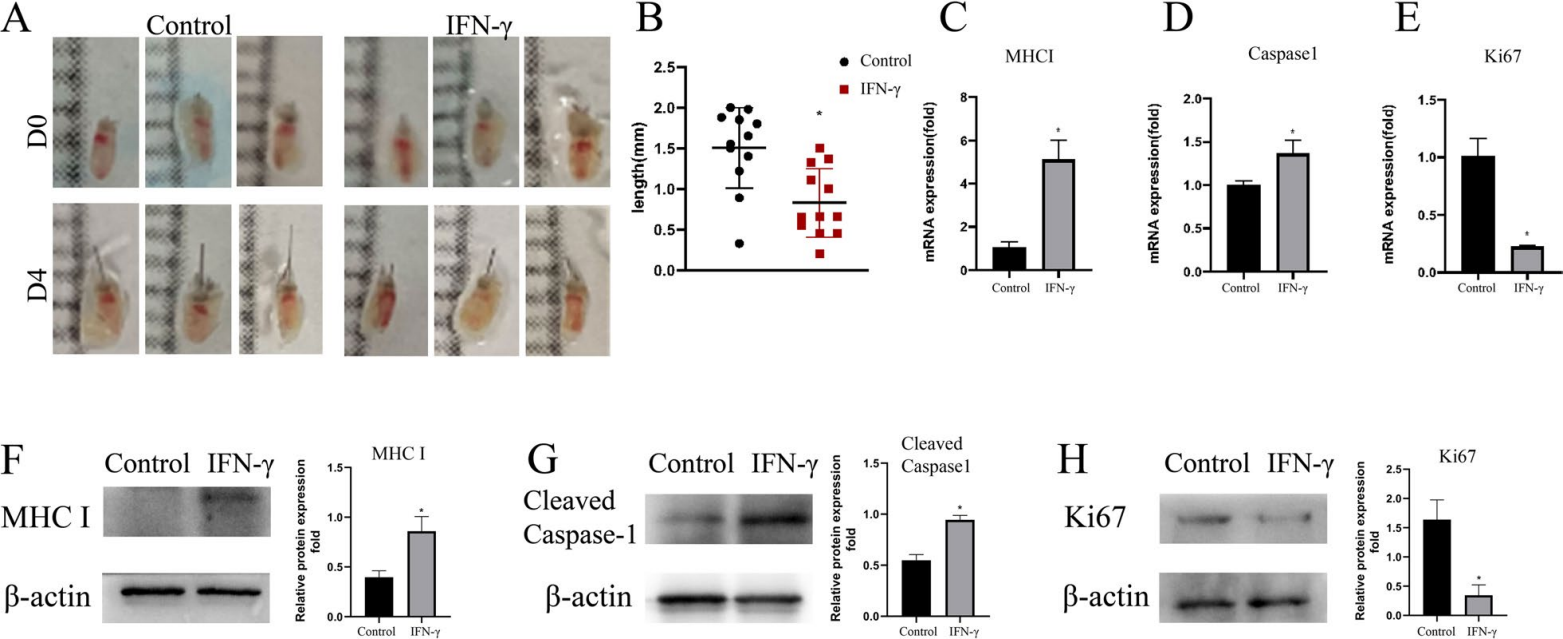
Growth-suppression and MHC I expression upregulation of interferon (IFN)-γ treatment in the organ-cultured model. Mouse vibrissa follicles were isolated and cultured in HF medium with PBS or 100 IU mL^−1^ IFN-γ addition. **A** Mouse vibrissa follicles were photographed on day 0 and day 4. **B** Hair shaft growth was inhibited by interferon (IFN)-γ in organ-cultured mouse vibrissa follicles on the 4 days (*n* = 12/each group). **C–E** The mRNA transcript levels of MHC I, caspase1 and Ki67 in mouse vibrissa after 4 days of PBS or IFN-γ treatment measured by qRT-PCR, expression levels of those mRNA were normalized with GAPDH. **F–H** The protein expression levels of MHC I, caspase1 and Ki67 in mouse vibrissa after 4 days of PBS or IFN-γ treatment measured by western blotting. Relative protein expression levels were averaged from three groups of biology repeatedly and normalized with β-actin. The results were expressed as the Mean ± SD, *p < 0.05

Hair follicle organ in vitro culture has been used extensively in hair regeneration research because it is appropriate to isolate, and its hair shaft proliferates [27, 28]. IFN-γ expresses in AA patients’ lesional skin and contributes to destroying hair follicle immune privilege by upregulating MHC I expression, which correlated directly with AA occurrence [29]. It is reported that IFN-γ-upregulating of MHC I expression in hair follicles can induce experimental catagen in vitro*,* and the addition of 100 IU mL^−1^ produced significant impairment of hair shaft elongation [6]. Therefore, we established a mouse vibrissa follicles organ model in vitro. Our results confirmed that IFN-γ addition decreased the hair shaft growth within 4 days (Fig. 2A, B) and increased the MHC I expression level of mouse vibrissa follicles (Fig. 2C). Those pathological phenomena are consistent with the main pathogenesis in the C3H/HeJ mouse model with AA [7]. Furthermore, Ki67 mRNA expression levels were significantly inhibited, while caspase1 mRNA expression levels were significantly upregulated by IFN-γ treatment (Fig. 2D, E). The same result is shown in Western blot analysis (Fig. 2F, H). This finding implied that using IFN-γ induction could lead to mouse vibrissa AA-like symptoms.

### Co-culture with HF-MSC decrease AA-like symptoms in vibrissa follicle

**Fig. 3:**
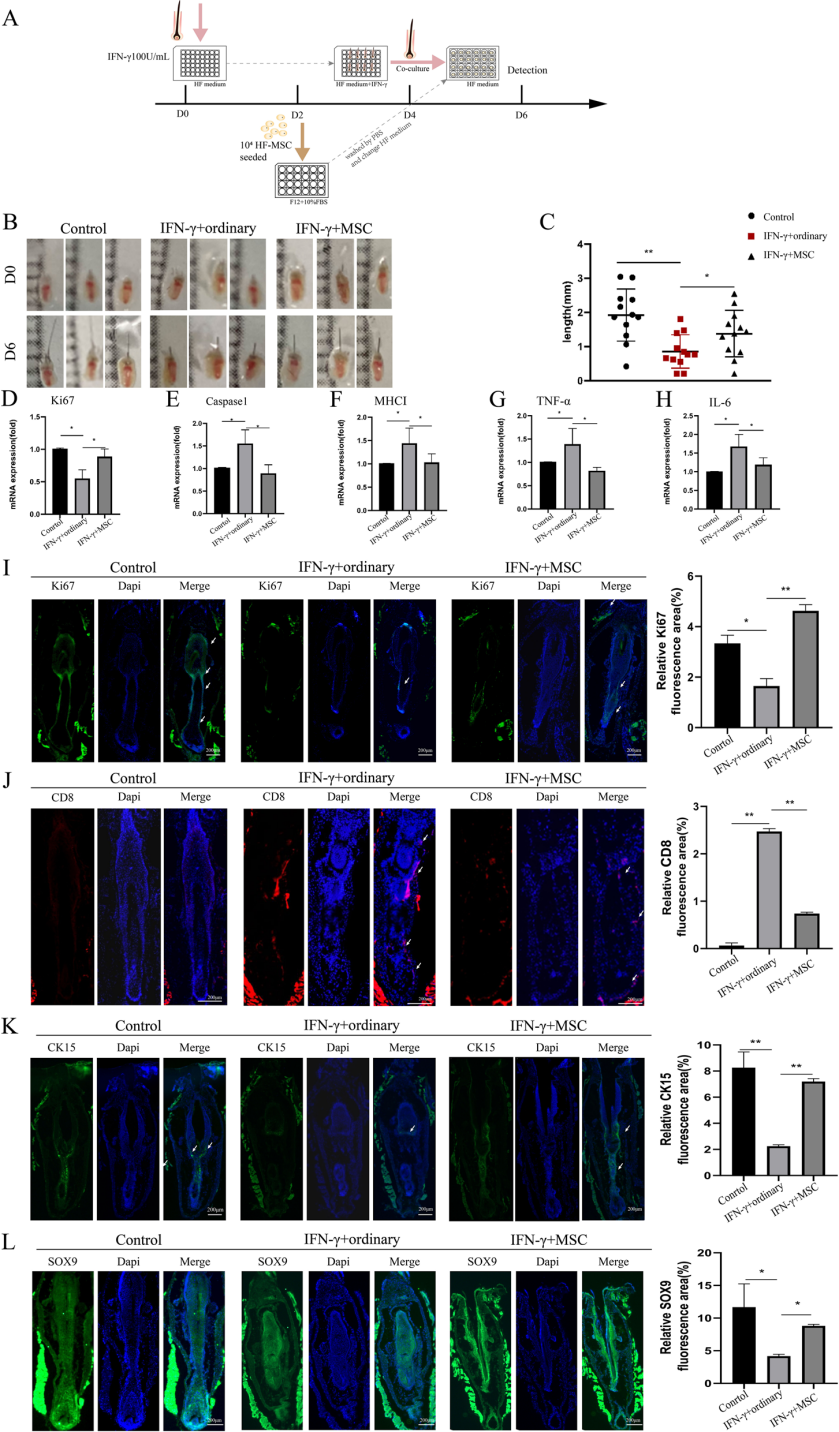
Co-culture with HF-MSC suppressed inflammation, increased hair shaft growth in IFN-γ treated mouse vibrissa follicles. Two groups (*n* = 12/group) of Mouse vibrissa follicles had been isolated and cultured with 100 IU mL^−1^ IFN-γ for 4 days than both groups changed HF medium and one group co-culture with HF-MSC for 2 days. And one group was cultured in an ordinary HF medium over 6 days as a control. **A** Illustration of co-culture with HF-MSC in IFN-γ induced mouse vibrissa follicles in vitro model. Hair shafts were measured on day 0 and day 6. **B–C** Co-culture with HF-MSC significantly enhance hair shaft length (*n* = 12). **D–H** The mRNA transcript levels of Ki67, caspase1, MHC I, TNF-α, IL-6 in mouse vibrissa were examined using qRT-PCR on day 6, expression levels of those mRNA were normalized with GAPDH. **I–L** Immunofluorescence staining on day 6 of mouse vibrissa follicles (bar = 200 μm), co-culture with MSC increased Ki67 expression (green) (**I**), and suppressed CD8 expression (red) (**J**). IFN-γ intervention reduced CK15 (**K**) and SOX9 (**L**) markers expression levels, HF-MSC treatment reversed the reduction of those two markers. Relative fluorescence areas were averaged from 6 to 8 fields. The results were expressed as the Mean ± SD, *p < 0.05

We then investigated whether HF-MSC treatment affects survival and immune regulation of mouse vibrissa follicles organ models. Two groups (*n* = 12/group) of mouse vibrissa follicles had been isolated and cultured with 100 IU mL^−1^ IFN-γ for 4 days, and then both groups changed ordinary medium and one group co-cultured with HF-MSCs for 2 days. Besides, the control group was cultured in the ordinarily defined medium over 6 days (Fig. 3A). Images of follicles were taken on day 0 and day 6 (Fig. 3B). IFN-γ induction retarded hair shaft growth, but this retardation was navigated by co-culture HF-MSC (Fig. 3C). Interestingly, the effect on co-culture with HF-MSC completely reversed the effect of IFN-γ treatment in gene and protein levels. HF-MSC treatment upregulated the expression of Ki67 (Fig. 3D) at the mRNA level and suppressed the mRNA expression of caspase1 and MHC I (Fig. 3E, F). TNF-α plays a critical role in the pathogenesis of certain autoimmune diseases [5], and AA is associated with the increase in levels of IL-6 [30]. The genes expression level for TNF-α (Fig. 3G) and IL-6 (Fig. 3H) decreased in co-culture with the HF-MSC group. Ki67 protein is closely related to cell proliferation, which exists during all active phases of the cell cycle but disappears from resting cells [31]. In co-culture with the HF-MSC group, we observed more fluorescent marked Ki67 protein (Fig. 3I). In AA patients’ skin, we identified significant infiltration of CD8^+^ T in and around hair follicles [29]. When the CD8 was marked with red, the CD8^+^ T cells were less observed in HF-MSCs treated mouse vibrissa follicles compared with the single cultivation (Fig. 3J). Cytokeratin (CK) 15 is the best marker for bulge stem cells in the hair and it is selectively throughout all stages of the hair cycle in different types of follicles [1]. The sex-determining region Y-Box 9 (SOX9) directs differentiation of the outer root sheath and is required to form hair stem cell compartment [32], while LIM Homeobox 2 (LHX2) is an essential factor in follicular organogenesis and cycling [33]. The immunofluorescent assay showed that IFN-γ intervention reduced CK15 (Fig. 3K) and SOX9 (Fig. 3L) markers expression, HF-MSC treatment reversed the reduction of those two markers, but dispensable for LHX2 (Additional file 1: Fig. S1).

### Injection of HF-MSC suppressed AA in C3H/HeJ mice

**Fig. 4:**
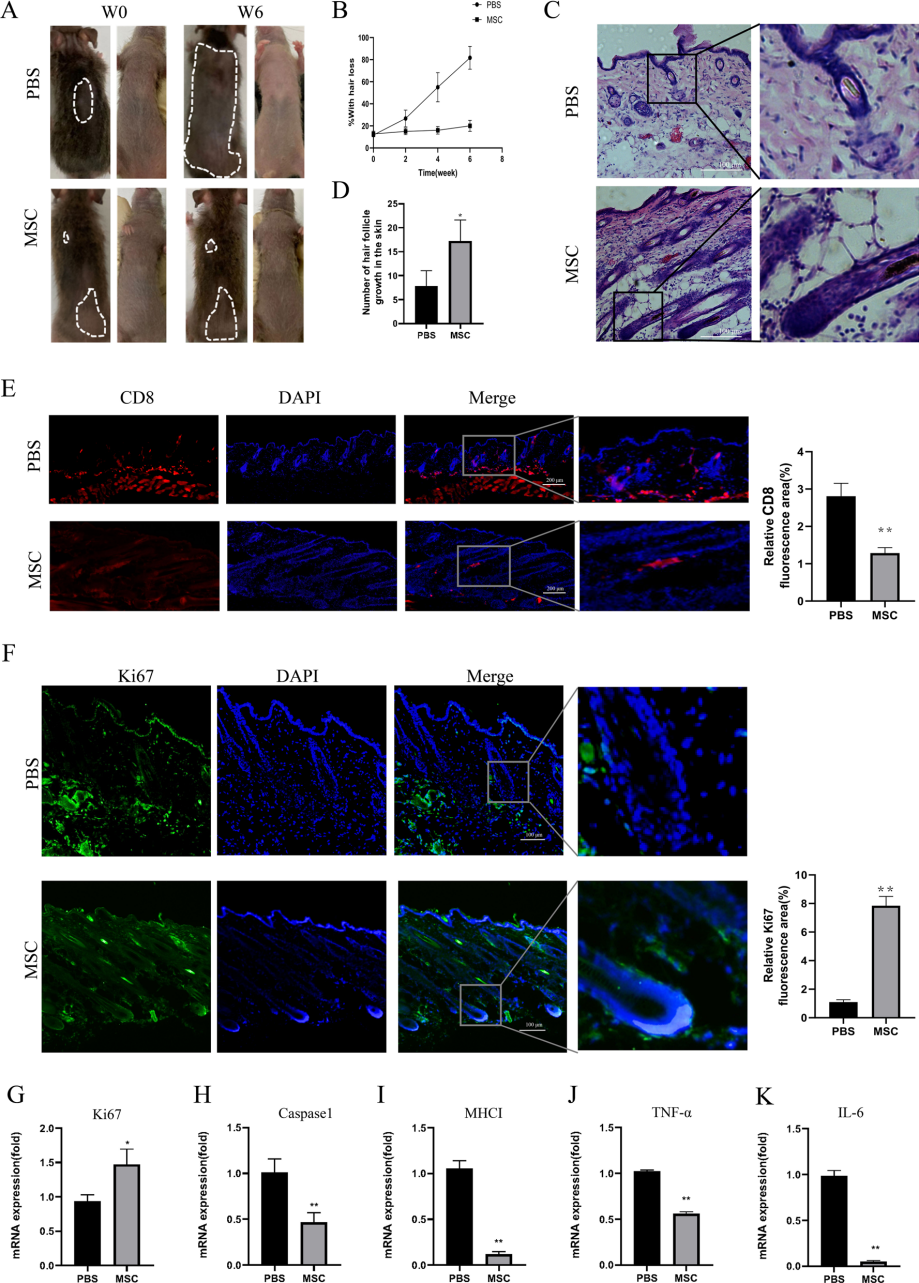
Injection of HF-MSC suppressed AA in C3H/HeJ mice. **A** Hair loss in C3H/HeJ mice intravenously injection of HF-MSC weekly. At 0 and 6 weeks after injection, the image of mice were photographed. **B** Compared with injected PBS (*n* = 4), mice did not continue to lose his hair that injected HF-MSC (*n* = 5). **C** H&E showed that HF-MSC-injected mice exhibited more hair follicles in anagen, while the PBS group had dystrophic hairs with lymphocyte infiltration (bar = 100 μm).The skin samples were harvested after 6 weeks treatment. **D** The number of hair follicle growth in the skin, which was averaged from 6 slides (10×). **E**–**F** HF-MSC-injection increased Ki67 expression (green) (bar = 100 μm) (**E**) and suppressed CD8 expression (red) (bar = 200 μm) (**F**) in C3H/HeJ skin. Relative fluorescence areas were averaged from 6 to 8 fields. **G-K** The mRNA transcript levels of caspase1, Ki67, MHC I, TNF-α, IL-6 in C3H/HeJ skin at 6 weeks after injection were examined using qRT-PCR, expression levels of those mRNA were normalized with GAPDH. The results were expressed as the Mean ± SD, *p < 0.05

Next, we evaluated whether HF-MSC could treat AA in vivo. HF-MSC was injected into the tail vein of the C3H/HeJ mice at 8–10 weeks after skin grafted (a time point that grafted C3H/HeJ mice with severe alopecia in the abdomen and starting to lose hair in the back). Mice treated with HF-MSC showed decreased hair loss compared to the PBS group (Fig. 4A). After 6 weeks, in the PBS group, the area of hair loss increased by 69.9%, whereas in the HF-MSC treated group was 12.6% more than the previous injection, however with intact abdominal hair (Fig. 4A, B). H&E-stained tissues revealed that MSC-injected mice exhibited more hair follicles in anagen, while the PBS group had dystrophic hairs with lymphocyte infiltration (Fig. 4C, D). Then, we investigated the lesional distribution to test whether HF-MSCs inhibit CD8^+^ T cell proliferation. As shown in Fig. 4E, the red-labeled CD8^+^ T immunofluorescent assay showed that the CD8^+^ T cells were less observed in the HF-MSC treated group than the PBS. Contrary, the ki67 was highly detected around HF in the HF-MSCs group and faint in the PBS group (Fig. 4F). RT-qPCR results revealed that, in HF-MSC group skin, Ki67 mRNA expression levels were upregulated, but gene expression related to apoptosis and damage of immune privilege was reduced, including caspase1, MHC I, TNF-α, and IL-6 (Fig. 4G–K).

## Discussion

The growing social pressure leads to poor physical and psychological health. Pressure is the important inducer of AA, which can cause deeper psychological problems, such as the high risk of neurotic personality, depression, anxiety, and deficiency [34], and some patients with suicidal tendencies have been also reported [5]. Corticosteroids are the most consensus achieved topical treatment in AA, but their side effects, short-term prescription condition, and limited age-group treatment still hinder it [12]. Considering safety and efficiency, more feasible therapies are required for this disease.

Some clinical studies have revealed alternative therapy options against hair loss. PRP has been proved to be medicative in regenerative plastic surgery and positively affects hair regrowth [35]. Local injection of autologous PRP upregulated AGA patients epidermal thickness, Ki67^+^ keratinocytes, and in the number of follicles [36]. Wingless-type (Wnt) signaling plays an important role in dermal papilla cell growth and is a key factor in stimulating hair growth. MSC transmit signaling and growth factors by platelets then platelets increase cell proliferation to lengthen the anagen phase, stimulate hair follicle development and suppress apoptotic cues [37]. Relative to the PRP, HF-MSC treatment shown lower injection frequency and more hair regrowth [38]. Gentile P. study the mechanical fragmented and centrifugated dermal adipose tissue-derived HF-MSC from scalp biopsy’s were autologous infiltrated on hair loss lesion [39], which indicated significant treatment in androgenic alopecia patients. But clinical studies for AA therapy are limited.

The preclinical study indicated that short-term IL-7 receptors in combination with low doses of Treg-tropic cytokines increased therapeutic effects in AA treatment [40]. The Janus kinase (JAK) family protein mediates IFN-γ receptors and γc family receptors signal. L. Xing et al. investigated that oral gavage or osmotic minipumps JAK inhibitors reduced effectors of the IFN-γ and γc cytokine receptors, blocked out the IFN signal, and prevented the development of AA. The local delivery JAK inhibitors promoted hair regrowth [41].

MSCs have remarkable therapeutic effects on the immune disease by reducing lymphocyte infiltration and releasing proinflammatory cytokines to promote the survival of damaged tissues [42–44]. The mechanism of MSC therapeutic function includes paracrine activity, transfer of mitochondria, and transfer of exosomes [42]. Dermal papilla cells co-cultured with human embryos mesenchymal stem cells have a higher propagation rate [45], depicting that MSC might promote hair growth. We confirm this phenomenon using a more accessible MSC for a cultural experiment and C3H/HeJ hair loss model treatment. Moreover, an MSC large-scale culturing technique (the United States Patent Application Number 13/517,068) generated HF-MSC in large numbers by using microspheres and spinning bottles, providing sufficient quantity for treatment supply.Bone marrow-derived MSC (BMSC) decreases AA incidence by inhibiting IFNG, Chemokine Ligand 1 Protein (CXCL) 9, and CXCL10 production and reducing CD8^+^NKG2D^+^ T cell infiltration [46]. Furthermore, BMSC and MSC-conditioned medium injection led hair follicles to be translated from telogen to anagen and enhanced proliferation of HFSCs positive for Krt15 and Sox9 [47]. In our study, we explored HF-MSC therapeutical effect in vitro using AA hair follicle organ model and in an AA mouse model.

AA is associated with CD8^+^ T cell infiltration and MHC I upregulation [48]. Therefore, we establish the AA model in vitro, which is the IFN-γ induced mouse vibrissa follicle with excessive CD8 and MHC I expression (Fig. 2). We observed that mouse vibrissa follicle co-culture with HF-MSC leads to immunotoxicity suppression and hair shaft elongation (Fig. 3). And HF-MSC treatment decreased AA mouse hair loss and reduced inflammation around HF in vivo (Fig. 4).

AA patients have higher IL-6 and TNF-α levels [49]. IL-6 suppressed HFSCs in a quiescent through JAK-STAT signaling (activate HFSCs to renew damaged hair follicles) [24]. TNF-α has an essential role in AA, but in clinical trials, TNF inhibitor treatments are contentious [50]. Also, adalimumab (a TNF-α inhibitor) effectively treating a case of alopecia Universalis (a severe AA stage) prove it again that TNF-α negative regulation is effective in the treatment of AA [51]. In this study, we observed that HF-MSCs treated hair follicles retained unbroken morphology is might benefit from negatively regulated IL-6 and TNF-α expression levels (Figs. 3, 4).

## Conclusion

Developing new therapeutic approaches that reverse multiple diseases is of great importance; however, AA treatment is still a challenge in the clinic. Our findings showed that HF-MSC promoted hair follicles proliferation and reduced HF inflammation as CD8^+^ T cells were less observed in HF-MSC treated Vibrissa follicles. Moreover, in the C3H/HeJ mice models, HF-MSC injection suppressed AA and inhibited immune privilege. Here we provide a potential therapeutic method for AA treatment, which is promising and beneficial for AA patients.

## Supplementary Information

**Additional file 1: Fig. S1**. IFN-γ intervention and MSC treatment not change Lhx2 expression (bar = 200 μm). Relative fluorescence areas were averaged from 6 slides. The results were expressed as the Mean ± SD, *p < 0.05.

## Data Availability

All relevant data and materials are available from the authors upon reasonable request.

## Abbreviations

AA: Alopecia areata
CD: Cluster of differentiation
MSC: Mesenchymal stem cells
HF-MSC: Hair follicle-derived MSC
HF: Hair follicles
DMEM/F12: Dulbecco’s Modified Eagle Medium/Nutrient Mixture F-12
FBS: Fetal bovine serum
MHC: Major histocompatibility complex
DAPI: 4′,6-Diamidino-2-phenylindole
IL: Interleukin
ANOVA: Analysis of variance
bFGF: Basic fibroblast growth factor
MHC: Major histocompatibility complex
HFSCs: Hair follicle stem cells
IL-6: Interleukin-6
TNF-α: Tumor necrosis factor-α
CXCL: Chemokine (C-X-C motif) ligand
H&E: Hematoxylin and eosin
ADSVCs: Adipose-derived stromal vascular cells
IFN: Interferon
JAK: Janus kinase
CK15: Cytokeratin 15
SOX9: Sex-determining Region Y-Box 9
Lhx2: LIM Homeobox 2
AGA: Androgenetic alopecia
PRP: Platelet-rich plasma
Wnt: Wingless-type

## References

[1] Al-Refu K (2012). Stem cells and alopecia: a review of pathogenesis. Br J Dermatol.

[2] Lee S, Lee WS (2017). Management of alopecia areata: updates and algorithmic approach. J Dermatol.

[3] Safavi K (1992). Prevalence of alopecia areata in the First National Health and Nutrition Examination Survey. Arch Dermatol.

[4] Lee HH, Gwillim E, Patel KR, Hua T, Rastogi S, Ibler E (2020). Epidemiology of alopecia areata, ophiasis, totalis, and universalis: a systematic review and meta-analysis. J Am Acad Dermatol.

[5] Toussi A, Barton VR, Le ST, Agbai ON, Kiuru M (2021). Psychosocial and psychiatric comorbidities and health-related quality of life in alopecia areata: a systematic review. J Am Acad Dermatol.

[6] Petukhova L, Duvic M, Hordinsky M, Norris D, Price V, Shimomura Y (2010). Genome-wide association study in alopecia areata implicates both innate and adaptive immunity. Nature.

[7] Chan LS, Vanderlugt CJ, Hashimoto T, Nishikawa T, Zone JJ, Black MM (1998). Epitope spreading: lessons from autoimmune skin diseases. J Invest Dermatol.

[8] Ito T, Ito N, Bettermann A, Tokura Y, Takigawa M, Paus R (2004). Collapse and restoration of MHC class-I-dependent immune privilege: exploiting the human hair follicle as a model. Am J Pathol.

[9] Philpott MP, Sanders DA, Bowen J, Kealey T (1996). Effects of interleukins, colony-stimulating factor and tumour necrosis factor on human hair follicle growth *in vitro*: a possible role for interleukin-1 and tumour necrosis factor-alpha in alopecia areata. Br J Dermatol.

[10] Ito T, Tokura Y (2014). The role of cytokines and chemokines in the T-cell-mediated autoimmune process in alopecia areata. Exp Dermatol.

[11] Jalili RB, Kilani RT, Li Y, Khosravi-Maharlooie M, Nabai L, Wang EHC (2018). Fibroblast cell-based therapy prevents induction of alopecia areata in an experimental model. Cell Transpl.

[12] Meah N, Wall D, York K, Bhoyrul B, Bokhari L, Sigall DA (2020). The Alopecia Areata Consensus of Experts (ACE) study: results of an international expert opinion on treatments for alopecia areata. J Am Acad Dermatol.

[13] Gentile P, Garcovich S (2020). Autologous activated platelet-rich plasma (AA-PRP) and non-activated (A-PRP) in hair growth: a retrospective, blinded, randomized evaluation in androgenetic alopecia. Expert Opin Biol Ther.

[14] Uccelli A, Moretta L, Pistoia V (2008). Mesenchymal stem cells in health and disease. Nat Rev Immunol.

[15] Bartholomew A, Sturgeon C, Siatskas M, Ferrer K, McIntosh K, Patil S (2002). Mesenchymal stem cells suppress lymphocyte proliferation *in vitro* and prolong skin graft survival *in vivo*. Exp Hematol.

[16] Anderi R, Makdissy N, Azar A, Rizk F, Hamade A (2018). Cellular therapy with human autologous adipose-derived adult cells of stromal vascular fraction for alopecia areata. Stem Cell Res Ther.

[17] Zhang X, Wang Y, Gao Y, Liu X, Bai T, Li M (2013). Maintenance of high proliferation and multipotent potential of human hair follicle-derived mesenchymal stem cells by growth factors. Int J Mol Med.

[18] Liu F, Shi J, Zhang Y, Lian A, Han X, Zuo K (2019). NANOG attenuates hair follicle-derived mesenchymal stem cell senescence by upregulating PBX1 and activating AKT signaling. Oxid Med Cell Longev.

[19] Liu JY, Peng HF, Gopinath S, Tian J, Andreadis ST (2010). Derivation of functional smooth muscle cells from multipotent human hair follicle mesenchymal stem cells. Tissue Eng Part A.

[20] Zhang X, Tang H, Mao S, Li B, Zhou Y, Yue H (2020). Transplanted hair follicle stem cells migrate to the penumbra and express neural markers in a rat model of cerebral ischaemia/reperfusion. Stem Cell Res Ther.

[21] Sun X, Gao Y, Chen H, Yang N, Zhang Y, Liu Q (2021). From hair to pancreas: transplanted hair follicle mesenchymal stem cells express pancreatic progenitor cell markers in a rat model of acute pancreatitis. Am J Transl Res.

[22] Liu Z, Lu SJ, Lu Y, Tan X, Zhang X, Yang M (2015). Transdifferentiation of human hair follicle mesenchymal stem cells into red blood cells by OCT4. Stem Cells Int..

[23] Wu C, Liu F, Li P, Zhao G, Lan S, Jiang W (2015). Engineered hair follicle mesenchymal stem cells overexpressing controlled-release insulin reverse hyperglycemia in mice with type L diabetes. Cell Transpl.

[24] Wang ECE, Dai Z, Ferrante AW, Drake CG, Christiano AM (2019). A subset of TREM2(+) dermal macrophages secretes oncostatin m to maintain hair follicle stem cell quiescence and inhibit hair growth. Cell Stem Cell..

[25] McElwee KJ, Boggess D, King LE, Sundberg JP (1998). Experimental induction of alopecia areata-like hair loss in C3H/HeJ mice using full-thickness skin grafts. J Invest Dermatol.

[26] Ito T, Ito N, Saathoff M, Bettermann A, Takigawa M, Paus R (2005). Interferon-gamma is a potent inducer of catagen-like changes in cultured human anagen hair follicles. Br J Dermatol.

[27] Jindo T, Tsuboi R, Imai R, Takamori K, Rubin JS, Ogawa H (1994). Hepatocyte growth factor/scatter factor stimulates hair growth of mouse vibrissae in organ culture. J Invest Dermatol.

[28] Robinson M, Reynolds AJ, Jahoda CA (1997). Hair cycle stage of the mouse vibrissa follicle determines subsequent fiber growth and follicle behavior *in vitro*. J Invest Dermatol.

[29] Dai Z, Xing L, Cerise J, Wang EH, Jabbari A, de Jong A (2016). CXCR3 blockade inhibits T cell migration into the skin and prevents development of alopecia areata. J Immunol.

[30] Bodemer C, Peuchmaur M, Fraitaig S, Chatenoud L, Brousse N, De Prost Y (2000). Role of cytotoxic T cells in chronic alopecia areata. J Invest Dermatol.

[31] Scholzen T, Gerdes J (2000). The Ki-67 protein: from the known and the unknown. J Cell Physiol.

[32] Vidal VP, Chaboissier MC, Lutzkendorf S, Cotsarelis G, Mill P, Hui CC (2005). Sox9 is essential for outer root sheath differentiation and the formation of the hair stem cell compartment. Curr Biol.

[33] Bhattacharya S, Wheeler H, Leid M, Ganguli-Indra G, Indra AK (2015). Transcription factor CTIP2 maintains hair follicle stem cell pool and contributes to altered expression of LHX2 and NFATC1. J Invest Dermatol.

[34] Mulinari-Brenner F (2018). Psychosomatic aspects of alopecia areata. Clin Dermatol.

[35] Gentile P, Garcovich S, Bielli A, Scioli MG, Orlandi A, Cervelli V (2015). The effect of platelet-rich plasma in hair regrowth: a randomized placebo-controlled trial. Stem Cells Transl Med.

[36] Gentile P, Cole JP, Cole MA, Garcovich S, Bielli A, Scioli MG (2017). Evaluation of not-activated and activated PRP in hair loss treatment: role of growth factor and cytokine concentrations obtained by different collection systems. Int J Mol Sci..

[37] Gentile P, Garcovich S (2019). Advances in regenerative stem cell therapy in androgenic alopecia and hair loss: Wnt pathway, growth-factor, and mesenchymal stem cell signaling impact analysis on cell growth and hair follicle development. Cells..

[38] Gentile P, Scioli MG, Bielli A, De Angelis B, De Sio C, De Fazio D (2019). Platelet-rich plasma and micrografts enriched with autologous human follicle mesenchymal stem cells improve hair re-growth in androgenetic alopecia. Biomolecular pathway analysis and clinical evaluation. Biomedicines..

[39] Gentile P (2019). Autologous cellular method using micrografts of human adipose tissue derived follicle stem cells in androgenic alopecia. Int J Mol Sci..

[40] Dai Z, Wang EHC, Petukhova L, Chang Y, Lee EY, Christiano AM (2021). Blockade of IL-7 signaling suppresses inflammatory responses and reverses alopecia areata in C3H/HeJ mice. Sci Adv..

[41] Xing L, Dai Z, Jabbari A, Cerise JE, Higgins CA, Gong W (2014). Alopecia areata is driven by cytotoxic T lymphocytes and is reversed by JAK inhibition. Nat Med.

[42] Spees JL, Lee RH, Gregory CA (2016). Mechanisms of mesenchymal stem/stromal cell function. Stem Cell Res Ther.

[43] Le Blanc K, Rasmusson I, Sundberg B, Götherström C, Hassan M, Uzunel M (2004). Treatment of severe acute graft-versus-host disease with third party haploidentical mesenchymal stem cells. Lancet (London, England).

[44] Ceccariglia S, Cargnoni A, Silini AR, Parolini O (2020). Autophagy: a potential key contributor to the therapeutic action of mesenchymal stem cells. Autophagy.

[45] Wu M, Sun Q, Guo X, Liu H (2010). hMSCs possess the potential to differentiate into DP cells *in vivo* and *in vitro*. Cell Biol Int Rep.

[46] Byun JW, Kim HJ, Na K, Ko HS, Song HJ, Song SU (2017). Bone marrow-derived mesenchymal stem cells prevent alopecia areata development through the inhibition of NKG2D expression: a pilot study. Exp Dermatol.

[47] Vidal VP, Chaboissier MC, Lützkendorf S, Cotsarelis G, Mill P, Hui CC (2005). Sox9 is essential for outer root sheath differentiation and the formation of the hair stem cell compartment. Curr Biol.

[48] Rückert R, Hofmann U, van der Veen C, Bulfone-Paus S, Paus R (1998). MHC class I expression in murine skin: developmentally controlled and strikingly restricted intraepithelial expression during hair follicle morphogenesis and cycling, and response to cytokine treatment *in vivo*. J Invest Dermatol.

[49] Kanda N, Koto M, Hoashi T, Saeki H (2019). Case of alopecia areata during dupilumab treatment for atopic dermatitis. J Dermatol.

[50] Pichler WJ (2006). Adverse side-effects to biological agents. Allergy.

[51] Gorcey L, Gordon Spratt EA, Leger MC (2014). Alopecia universalis successfully treated with adalimumab. JAMA Dermatol.

