# Supplementary figures and images for: Hair follicle-derived mesenchymal stem cells decrease alopecia areata mouse hair loss and reduce inflammation around the hair follicle
Source: Stem Cell Res Ther. 2021 Oct 21;12:548. doi: 10.1186/s13287-021-02614-0 (PMC8532319; doi:10.1186/s13287-021-02614-0)

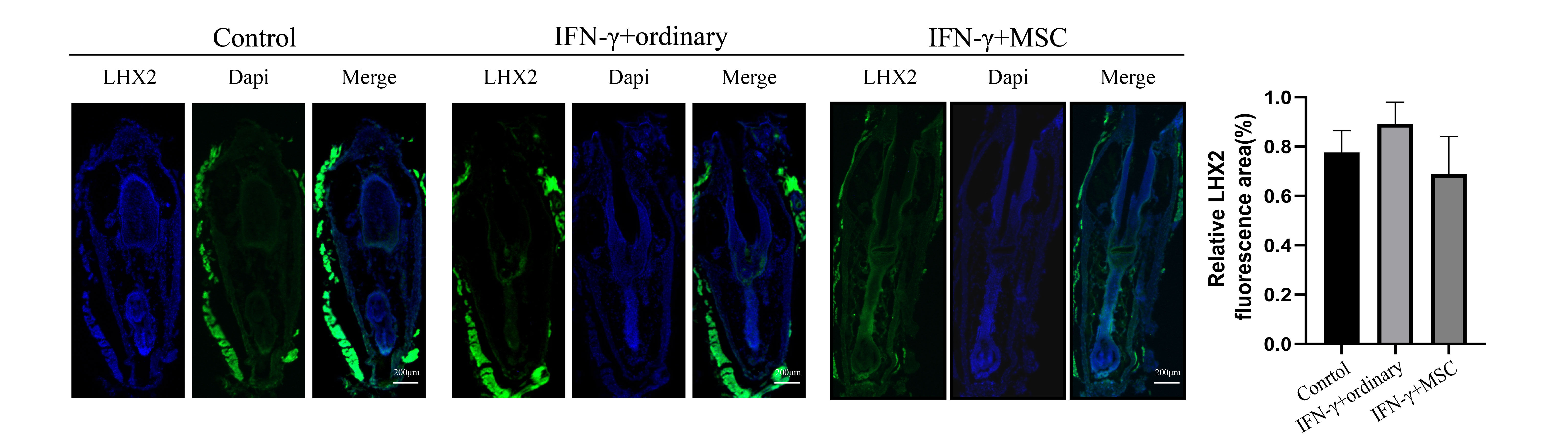

Supplement: Supplementary file 1 — Additional file 1: Fig. S1. IFN-γ intervention and MSC treatment not change Lhx2 expression (bar = 200 μm). Relative fluorescence areas were averaged from 6 slides. The results were expressed as the Mean ± SD, *p < 0.05. [file 13287_2021_2614_MOESM1_ESM.jpg]
